# Dielectrophoretic behaviours of microdroplet sandwiched between LN substrates

**DOI:** 10.1038/srep29166

**Published:** 2016-07-07

**Authors:** Lipin Chen, Shaobei Li, Bolin Fan, Wenbo Yan, Donghui Wang, Lihong Shi, Hongjian Chen, Dechao Ban, Shihao Sun

**Affiliations:** 1 School of Materials Science and Engineering, Hebei Engineering Laboratory of Photoelectronic Functional Crystals, Hebei University of Technology, Tianjin 300130, China; 2 Tianjin Urban Construction Institute, Tianjin 300384, China

## Abstract

We demonstrate a sandwich configuration for microfluidic manipulation in LiNbO_3_ platform based on photovoltaic effect, and the behaviours of dielectric microdroplet under this sandwich configuration are investigated. It is found that the microdroplet can generate in the form of liquid bridge inside the LiNbO_3_-based sandwich structure under the governing dielectrophoretic force, and the dynamic process of microdroplet generation highly depends on the substrate combinations. Dynamic features found for different combinations are explained by the different electrostatic field distribution basing on the finite-element simulation results. Moreover, the electrostatic field required by the microdroplet generation is estimated through meniscus evolution and it is found in good agreement with the simulated electrostatic field inside the sandwich gap. Several kinds of microdroplet manipulations are attempted in this work. We suggest that the local dielectrophoretic force acting on the microdroplet depends on the distribution of the accumulated irradiation dosage. Without using any additional pumping or jetting actuator, the microdroplet can be step-moved, deformed or patterned by the inconsecutive dot-irradiation scheme, as well as elastically stretched out and back or smoothly guided in a designed pass by the consecutive line-irradiation scheme.

Lithium niobate (LN) is regarded as a potential substrate material for integrated optics industry due to its strong electro-optical, pyroelectric, piezoelectric and nonlinear optical properties. As LN shows good biocompatibility, many biomedical functions based on photonics are expected to be integrated on LN chips. In such a way, lab-on-chips may open new horizons on biological analysis^1^,^2^,^3^,^4^,^5^, clinical diagnostics^6^,^7^,^8^ and drug discovery^9^,^10^,^11^. For example, T. Yang and P. Minzioni *et al*. proposed a novel optofluidic design for real-time sorting based on the cell mechanical properties. The sorting component can be realized on LN substrates with many other functional units (e.g. waveguide and SAW actuator) integrated for cell counting and alignment^1^,^2^,^3^. Among various integrated functions, the most important one required by the LN-based lab-on-chip is the manipulation of microdroplets, which is the key technique for reducing both reactant consumption and reaction time.

In the past few years, a couple of droplet-based microfluidic systems have been proposed basing on photovoltaic effect^12^,^13^,^14^,^15^,^16^,^17^,^18^,^19^,^20^,^21^,^22^,^23^. A. C. Muir *et al*.^12^ first reported the change of inherent wetting property of LN crystal surface under UV irradiation. Their results clearly show that photo-excited carriers might be used for arranging microdroplets. Later, M. Esseling and C. Denz *et al*. made a throughout investigation on dielectrophoretic (DEP) trapping in two-dimensional patterns on LiNbO_3_:Fe (LN:Fe) crystals^13^,^14^,^15^,^16^,^17^. While M. Carrascosa *et al*. studied the DEP trapping both in experimental and theoretical aspects, and they established numerical algorithm to predict the trapped object distribution^18^,^19^,^20^,^21^,^22^,^23^. Recently, an optofluidic droplet router based on photorefractive effect has been subtly fabricated by Esseling *et al*., who used DEP force created by the bulk photovoltaic effect to route and steer microdroplets on LN substrate, realizing the microfluidic manipulation in a LN-based lab-on-chip^17^ for the first time.

The DEP force originates from the unequal electrostatic force of polarization charges induced on dielectrics when they are subjected to nonuniform electric fields^24^, and it has been widely used for the manipulation of microfluidics and micron-sized particles such as biological droplets and cells^25^,^26^. Thomas *et al*. presented a novel particle-trapping design by using negative DEP to trap cells in high conductivity physiological media^25^. Kumar *et al*. showed many kinds of opto-electric manipulations in microfluidics that utilized DEP force in their review^26^. For most of the above designs, electrodes have to be fabricated on the substrates^25^,^26^,^27^,^28^, which certainly increases the complexity of the devices. For photovoltaic materials such as LN:Fe crystals, DEP force can be created directly in the substrate through photovoltaic current^29^ (without using electrodes), which enables more flexible and advantageous devices fabricated possibly.

The previous microdroplet manipulation usually employed one LN substrate. For example, in Esseling’s wrok^17^ channel structure was made of PDMS and LN was used as the bottom of the channels. Through the photovoltaic effect of the LN substrate the incident illumination pattern was transformed into electric field distributions. Droplet, actuated by the pumping component, moved inside channel structure and this electric field distribution was responsible for the droplet manipulations. However, with the development of lab-on-chip, more complicated configuration is very likely to be required. In this paper, we demonstrate a sandwich configuration based on LN substrates. The sandwich structure allows the microdroplets to be generated in the form of liquid bridge and move in the gap of two LN substrates under the combined actions of the two substrates. The microdroplets sandwiched between two LN substrates will be affected by the electrostatic forces generated on both substrates simultaneously. In this case, the manipulation force could be enhanced by the proper configuration of double substrates, and consequently the droplet can be manipulated actively in multimode with higher efficiency. Thus, pumping or jetting component can be removed from the microfluidic system, which is often required by an isolated design. As the microdroplet behaviours under the combined actions of the two substrates haven’t been reported yet until now, the study regarding this issue is useful not only to the novel design of future LN-based lab-on-chip but also to the understanding of the kinetic mechanism of electrified liquid on the LN substrate.

## Experimental Procedures

**Figure 1:**
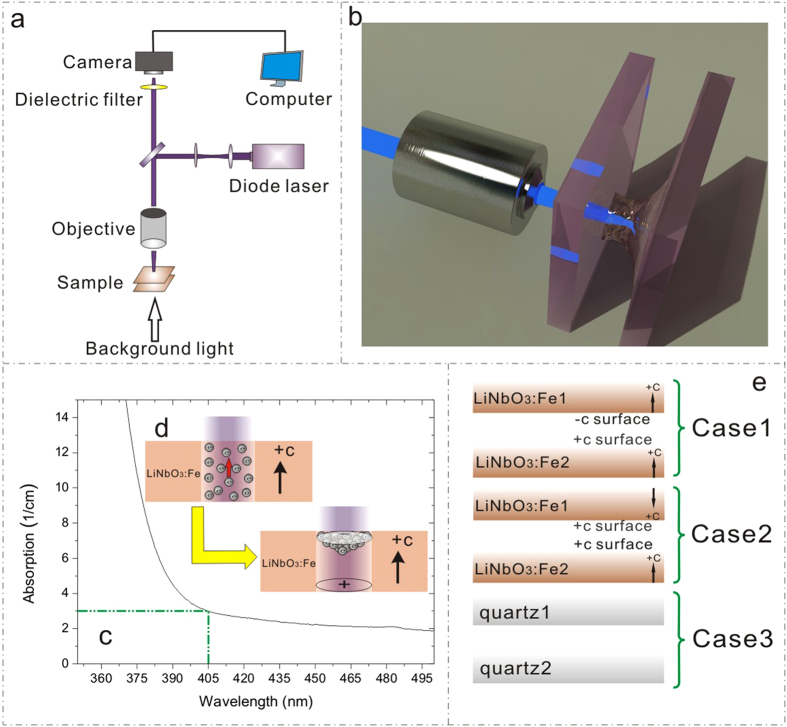
(**a**) The experimental setup for observing the behaviours of oil microdroplet under sandwich configuration; (**b**) 3D view of the microdroplet manipulation in the sandwich configuration; (**c**) UV-Vis absorption spectra of LN:Fe samples used in our work and the absorption at 405 nm is measured to be around 3.0 cm^−1^. (**d**) The photovoltaic mechanisum of the LN:Fe crystal: under the irradiation, the photo-excited electrons in Fe-doped LN are transported along the c axis, forming an photovoltaic current toward +c surface. This effect makes negative charges accumulated in the +c surface and positive charges left in the −c surface. (**e**) Three combinations of sandwich substrates in our work: two LN:Fe substrates with +c to −c surface (Case 1), two LN:Fe substrates with +c to +c-surface (Case 2), and two quartz substrates (Case 3) as a control experiment.

The sandwich substrates used in our experiment consist of two c-cut congruent LN:Fe crystals (doped with 0.03 wt% Fe_2_O_3_). The thickness of each sample is about 0.5 mm. The experimental setup for observing the behaviours of oil microdroplet under sandwich configuration is shown in Fig. 1(a). The laser beam (405 nm, CniLaser) was focused by a microscope objective on the sandwich structure, and the bottom substrate was used as the base of a liquid reservoir. A camera was used for capturing the dynamic process with the white light used as a background. The two substrates were fixed on two linear motorized stages where the grating rulers were equipped (not shown in Fig. 1(a)). The distance between the two sandwich substrates can be adjusted by the motion controller. In our work, two combinations of sandwich substrates were adopted (Fig. 1(e)): +c to −c-surface (Case 1) and +c to +c-surface (Case 2). Distinct behaviours of the dielectric liquid were observed in the two cases. In order to rule out the possibility that the liquid behaviours are due to the laser-induced heating of dielectric liquid, another combination of two quartz substrates (Case 3) was also tried as a control experiment.

Sample preparation and experimental procedures are as follows: substrates were firstly sonicated in ethanol for 20 min, and then in deionized water for 20 min. After cleaning and drying, dielectric liquid (transformer oil) was spread out as a thin film on the bottom substrate. Then, two substrates were attached to linear motorized stages, with the distance between them being adjusted to be about 25 μm. When the laser beam is applied to the substrates, the oil film will be governed by DEP force, and the microdroplet can be generated in the form of liquid bridge and manipulated in multimode in the gap of the two substrates. In our experiment, the medium in the gap (outside the oil drop) is air.

## Results and Discussion

### The general principle

Dopant Fe can introduce electron traps (Fe_Li_^2+/3+^) in the LN lattice and induce a strong absorption in the Visible range. Figure 1(c) shows the UV-Vis absorption spectra of LN:Fe samples used in this work and the absorption at 405 nm was measured to be around 3.0 cm^−1^. Under the illumination, the electrons can be easily photo-excited from Fe^2+/3+^ traps and they may be transported by both diffusion and photovoltaic fields^30^. In LN:Fe, the dominating charge transport mechanism is the photovoltaic (or photogalvanic) effect^29^. The strong anisotropy of this effect results in much larger photovoltaic currents parallel to the polar c axis than perpendicular to it (see Fig. 1(d)). The photovoltaic current density (J_pv_) can be described by J_pv_ = GαI, where G is the Glass constant, α the absorption and I the laser intensity^29^,^31^,^32^. Under the illumination, the photo-excited electrons are easily accumulated in the +c surface of LN:Fe, leaving a large amount of positive charges in the −c surface (see Fig. 1(d)). The preferential accumulation of photo- excited electrons in +c surface has been used for selective Ag nano-particle deposition^33^ or proton-exchange on LN surface^34^. Due to the accumulation of photo-excited electrons, strong inhomogeneous electrostatic field comes into being at the LN surface. Under this electrostatic field, the dielectric liquid will be polarized, and moves, governed by the DEP force, toward the place where the maximum of the electrostatic field is located^35^. The continuing influx of the dielectric liquid will raise the local liquid level and lead to the rounded liquid meniscus. If the two substrates are sufficiently close, the rounded liquid meniscus may touch the top substrate and a microdroplet generates in the form of liquid bridge (see Fig. 1(b)).

### Microdroplet generation

**Figure 2:**
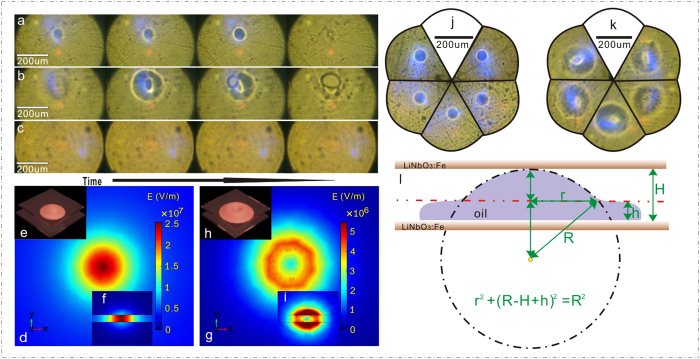
(**a–c**) The dynamic processes of the microdroplet generation in the Case 1 (**a**), Case 2 (**b**) and Case3 (**c**). (**d**–**i**) The simulation results of the electrostatic field (vector norm) distribution inside the gap for the Case 1 (**d**,**f**) and Case 2 (**g**,**i**). (**d**–**i**) Corresponding to the top view (x-y plane) (**d**,**g**) and the side view (z-y plane) (**f**,**i**). Herein, the z-axis is parallel with the c-axis of LN. (**e**,**h**) The peak-like (**e**) and volcano-like (**h**) profiles of the liquid meniscus in the Case 1 and Case 2, respectively. (**j**,**k**) The five results selected randomly in the Case 1 (**j**) and Case 2 (**k**). (**l**) The geometrical relationship between the parameters of rounded meniscus in the Case 1.

The two combinations of c-cut Fe-LN substrates were tried: +c to −c surface (Case 1) and +c to +c surface (Case 2). We found that the dynamic processes of microdroplet generation under the same irradiation intensity are quite different in these two cases. As shown in Fig. 2(a) for the Case 1, a solid circle appears at the laser spot, and it becomes increasingly obvious and finally turns into a microdroplet (in the form of liquid bridge). However, in the Case 2 (see Fig. 2(b)) concentric circles rather than a solid circle are observed before the microdroplet formation. The existence of concentric circles indicates the complexity of the microdroplet generation in the Case 2. For comparison, the result of two quartz substrates (Case 3) was shown in Fig. 2(c). Neither solid nor concerntric circles are observed, revealing that the dielectric liquid behaviours are indeed connected with the photo-induced effect of LN:Fe substrates. In order to check the statistical significance of the distinct behaviours in the Case 1 and Case 2, we tried the microdroplet generation tens of times and found that the experimental reproducibility is quite good. Figure 2 (j,k) show five results selected randomly for the Case 1 and Case 2, respectively. The statistical significance (P < 0.05) can be guaranteed in our experiments.

**Figure 3:**
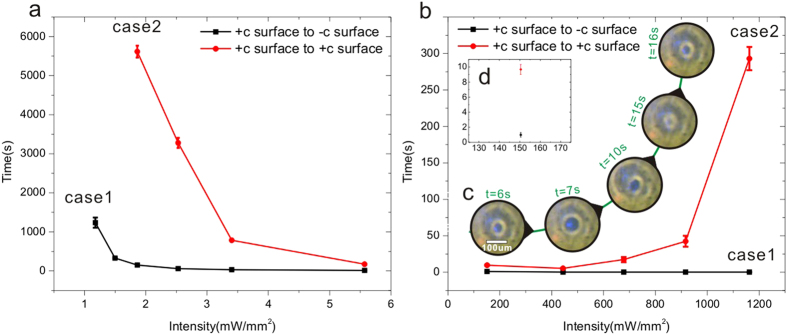
The dependences of the accumulation duration δt on the average intensity I_A_ for both cases at low (**a**) and high (**b**) intensity ranges. The standard error of mean is given for all data in both figures. The inset image (**c**) shows the repeated up-and-down (vibration-like) movement of the oil at I_A_ = 674.2 mW/mm^2^. The inset figure (**d**) shows the data at I_A_ = 150 mW/mm^2^ in a magnified scale.

The features shown by the above results can be connected with the electrostatic field distribution. According to the photogalvanic effect^29^, photo-excited electrons accumulate in the illumination region of +c surface while the positive charges are left at −c surface^36^,^37^. Charges at each substrate surface can be regarded as an independent source of electrostatic field, and the behaviours of the dielectric liquid should be governed by the superposed field. In order to better understand the microdroplet behaviours in the sandwich structure, we simulated the electrostatic field (vector norm) in both cases by using the finite-element model. A 25-μm gap between the two substrates was used for simulations. Assuming the laser intensity at the focus follows the Gaussian distribution and neglecting the diffusion effect of the photo-exited electrons at the surface^37^, the surface charge density σ can be given by σ = δtJ_pv_ = δtGαI(x, y) = δtGαI_0_Exp[−2(x^2^ + y^2^)/ω^2^]^20^, where δt is the accumulation duration, I_0_ the maximum intensity and ω the spot size of the beam waist at the focus. It is worth noting that the incident laser power P equals to the integration of the I(x, y) over the illumination area and the average intensity I_A_ at the focus could be given approximately as I_A_ = P/πω^2^, where the ω is estimated to be 50 μm. The diffusion of the photo-exited electrons can be neglected if the microdroplet generation is completed in very short time, i.e. the accumulation duration δt for photo-excited electrons is quite short. As shown in Fig. 3(b), the microdroplet generation is finished in a short δt of 1 s at the illumination condition of I_A_ = 150 mW/mm^2^ (or P = 1.18 mW) in the Case 1. The Glass constant at 405 nm is estimated to be 3.3 pAcm/mW^31^, and the absorption α at 405 nm is measured to be approximately 3.0 cm^−1^. Figure 2(d,f,g,i) show the simulation results of the electrostatic field (vector norm) distribution inside the gap by using the parameters: δt = 1 s, P = 1.18 mW, ω = 50 μm, G = 3.3 pAcm/mW and α = 3.0 cm^−1^ for the Case 1 and Case 2, respectively. Among them, (d), (g) and (f), (i) correspond to the top view (x-y plane) and the side view (z-y plane), respectively. Herein, the z-axis is parallel with the c-axis of LN. It can be clearly seen that the main difference between the two cases is the spatial position of the maximum of the electrostatic field: it is located at the center of the illuminated area in the Case 1 while it appears on the circle edge deviating from the center in the Case 2. As mentioned above, the rising of the liquid level at the location of the maximal electrostatic field may lead to the rounded liquid meniscus. In the Case 1, the rounded liquid meniscus at the center (peak-like profile in Fig. 2(e)) is observed as a solid circle in Fig. 2(a). However, in the Case 2 the rising of the liquid level happens at the circle edge (volcano-like profile in Fig. 2(h)) rather than at the center. Consequently, we observed the concentric circles before the final formation of liquid bridge.

Concerning the behaviours of electrified fluid, the most famous phenomena are about its meniscus evolution and the fluid jetting, which are firstly reported by G. Taylor *et al*.^38^. Recently, E.O. Elele *et al*. have studied the evolution of the drop meniscus from a rounded shape to a cone under the electrical field^39^. In their work, the balance of the capillary and electric pressures in the meniscus was used to give E_a_~(2γ/ε_0_R)^1/2^, where E_a_ is the electrostatic field in the air, γ the surface tension, R the radius of meniscus curvature and ε_0_ the dielectric constant of surrounding media^38^,^39^,^40^. Considering the geometry in the Case 1, the R could be calculated roughly through the relationship r^2^ + (R − H + h)^2^ = R^2^, given the known r, h and H. From Fig. 2(a,j), the r is estimated to be 27 μm in the Case 1. The H and h are about 25 and 8 μm. In addition, the γ of the transformer oil is estimated to be 0.04 N/m. By using the above parameters, E_a_ required by the microdroplet generation is calculated to be ~1.7 × 10^7^ V/m, which is in good agreement with the magnitude order of the simulated electrostatic field (1 ~ 2.5 × 10^7^ V/m) inside the gap. In fact, the photovoltaic open circuit field E_phv_ could easily reach the value of 10^7^ V/m, as already reported by many researchers^29^,^30^,^31^.

The difference between the Case 1 and 2 can also be reflected through the comparison of the time required by the microdroplet generation (i.e. the accumulation duration δt). The dependences of the accumulation duration δt on the average intensity I_A_ are shown in Fig. 3(a,b) for both cases. It can be seen in both figures that the microdroplet generation in the Case 1 requires much less time than that in the Case 2. As shown in Fig. 2, the electrostatic field (δt = 1 s) in the Case 1 is much higher than that in the Case 2. In other words, the accumulation duration of δt = 1 s is insufficient for the microdroplet generation in the Case 2. As a matter of fact, it takes δt = 9 s in the Case 2 to reach the electrostatic field required by the microdroplet generation.

Figure 3(a) shows the increasing irradiation intensity I_A_ can accelerate the microdroplet generation for both cases. For inducing the electrostatic field required by the microdroplet generation, a critical surface charge density has to be reached. Since the surface charge density σ is proportional to δtGαI, increasing the irradiation intensity may shorten the accumulation duration δt. However, Fig. 3(b), which contains the data in the range of high intensity, shows that the microdroplet generation in the Case 2 may be slowed down by the increase of the irradiation intensity I_A_. The inset image of Fig. 3(b) shows the dynamic process of the microdroplet generation (Case 2) under the intensity I_A_ of 674.2 mW/mm^2^. The microdroplet generation starts to slow down just under this intensity. It can be clearly seen that the inner one of the concentric circles changes its size repeatedly, prolonging the final formation of the liquid bridge. The unexpected slowing-down of the microdroplet generation in the Case 2 has to be connected to its special superposed electrostatic field.

**Figure 4:**
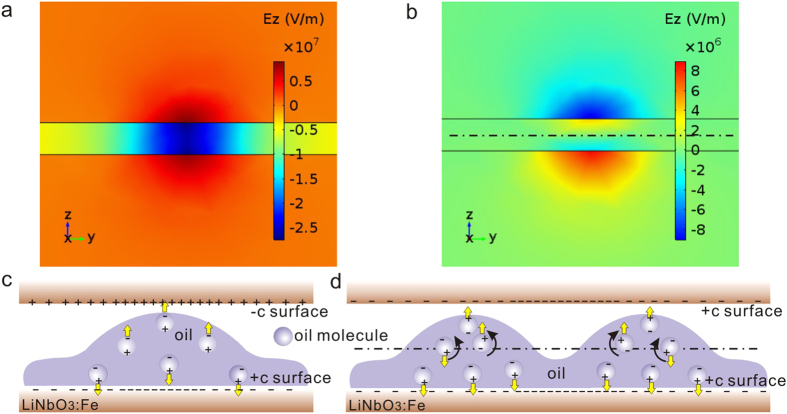
(**a,b**) The distribution of the electrostatic field (vertical component E_z_) in the side view (z-y plane) for the Case 1 (**a**) and Case 2 (**b**). Herein, the z-axis is parallel with the c-axis of LN. (**c**,**d**) The oil molecule polarization during the microdroplet generation in the Case 1 (**c**) and Case 2 (**d**). The electrostatic field in the Case 2 can be divided by the dot line into two parts having opposite directions.

If we just focus on the vertical component (E_z_) of the electrostatic field (see Fig. 4), one significant difference between the Case 1 and 2 can be found: the whole electrostatic field in the Case 1 has the same direction (see the blue region inside the gap in Fig. 4(a)) while the electrostatic field in the Case 2 can be divided by the dot line into two parts having opposite directions (see the slight orange and blue regions inside the gap in Fig. 4(b)). As mentioned above, the rising of the local liquid level may make a part of polarized oil in the blue region cross the dot line and enter the orange region. However, this part of oil will be affected by the push-back interaction of the reversed electrostatic field in the slight orange region before the oil polarization is fully aligned to the new electrostatic field direction. This competition may result in a repeated up-and-down (vibration-like) movement of the oil around the dot line. Moreover, the competition will be enhanced by the increased electrostatic field under the strong irradiation. In other words, the high irradiation intensity may bring the vibration-like movement to higher frequency, which certainly delays the formation of the stable oil bridge. As a matter of fact, the repeated variation of the inner circle size shown in Fig. 3(c) provides the best evidence for this vibration-like movement of oil. Therefore, the slowing-down of the microdroplet generation with the increasing irradiation intensity can only happen in the Case 2 but never in the Case 1. Another significant difference between the Case 1 and 2 is concerning the electrostatic fields in the LN substrates (E_s_) and in the air gap (E_a_). E_s_ is lower than E_a_ in the Case 1 while E_s_ is higher than E_a_ in the Case 2. In both cases, the direction of E_s_ is contrary to that of the photovoltaic current J_pv_. Higher E_s_ in the Case 2 may induce a drift current along the opposite direction of J_pv_, suppress the charge accumulation in the substrate surface^31^, and slow down the microdroplet generation under the high-intensity irradiation.

As the matter of fact, the relatively strong E_a_ in the double-LN-substrate structure (LN-LN structure) is due to the double contribution from the top and bottom LN substrates, and the manipulation force on the microdroplets in this structure is therefore much higher than that in single-LN-substrate structures (LN-glass or LN-plastic structures). In fact, the manipulation force exerted by single-LN-substrate has a limit due to the existence of the saturation field in LN:Fe^31^. This limit of the manipulation force is independent of the irradiation parameters such as intensity and duration. Thus, for further enhancing the manipulation force, another LN substrate has to be added. As compared with the single-LN- substrate structures, the double-LN-substrate structure is costly and difficult to be fabricated for lab-on-chip devices. However, we have to emphasize that the employment of double-LN-substrate structure can enhance the manipulation force and therefore benefit the multimodal and efficient microfluidic manipulation in lab-on-chip devices.

It should be noted that the intensity loss induced by the substrate absorption is not considered in the above simulations. In our case, one 0.5-mm-thick LN:Fe sample may cause the intensity loss of ~14%. If it is taken into account, the intensity reaching the top and bottom substrates will be different, and so will the light-induced surface charge densities of the two substrates. This difference (14%) may lead to an asymmetry of the electrostatic field, but it does not change the basic profile of the electrostatic field distribution. Therefore, any explanation made basing on the above simulation results is still reliable.

### Microdroplet manipulation

Since the microdroplet generation is more efficient in the Case 1 than in the Case 2, the configuration (+c to −c surface) of the Case 1 is adopted in this work for the further investigation about the microdroplet manipulation. Note that microdroplet manipulation in the Case 2 is more complicated and versatile. The special behaviours of the microdroplet in this case will be reported in the forthcoming papers.

Since the surface charge density σ is proportional to δtGαI, the accumulation duration δt contributes to the electrostatic field and the DEP force. The local DEP force on the microdroplet is determined by the spatial distribution of the surface charge density that is proportional to the accumulated irradiation dosage in the history. This is because the space charges, responsible for the DEP force, would not disappear immediately even after the laser is already switched off and its decay may last from tens of seconds to several hours depending on the local dark conductivity. Therefore, any past irradiation around the microdroplet will be partly preserved in the form of space charges, contributing to the local DEP force with its strength proportional to the irradiation dosage. In our experimental setup, the simplest method for controlling the irradiation dosage is to modify the accumulation duration δt, i.e. to change the irradiation time for dot-irradiation or to change the scanning speed for line-irradiation.

**Figure 5:**
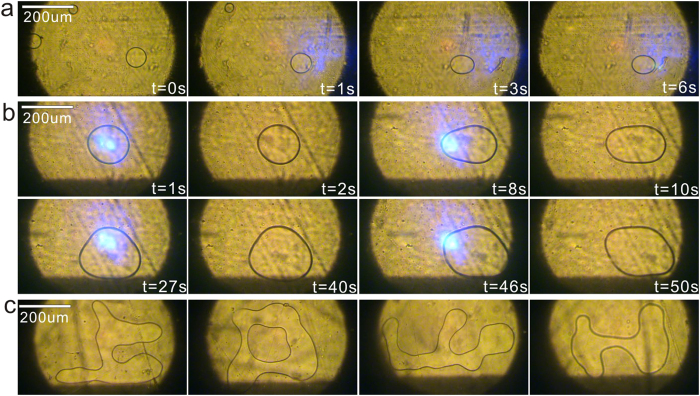
The dynamic processes of the microdroplet step-moving (**a**), deformation (**b**) and patterning (**c**) through the inconsecutive dot-irradiation scheme. In (**a**), three discrete dot-irradiations are preformed one by one around the microdroplet and their irradiation time is designed to increase gradually from 1 s to 3 s. In (**b**), the microdroplet is manipulated by four close dot-irradiations with the dot-irradiation time of ~1 s. In (**c**), the Chinese characters 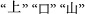 and English letter “H” are made through the same dot-irradiation scheme.

The inconsecutive dot-irradiation scheme is firstly adopted for microdroplet manipulation. In Fig. 5(a), three discrete dot-irradiations are preformed one by one around the microdroplet and their irradiation time is designed to increase gradually from 1 s to 3 s. In this case, the microdroplet is attracted by three irradiated dots sequentially and moves in a line step by step. Figure 5(b) shows the manipulation of microdroplet under four close dot-irradiations with the dot-irradiation time of ~1 s. It can be seen that the microdroplet is almost fixed at the original place but deformed from circle shape to parallelogram one. Through the same dot-irradiation scheme, more complicated microdroplet pattern can be achieved in Fig. 5(c), where the Chinese characters “” “” “” and English letter “H” are made in such a “microdrawing” way. The microdroplet manipulation effect (step-moving or drawing) of the dot-irradiation scheme depends on the distances between those discrete irradiated dots and their irradiation dosages. Closer dot spaces and irradiation dosages usually result in the “microdrawing” effect.

**Figure 6:**
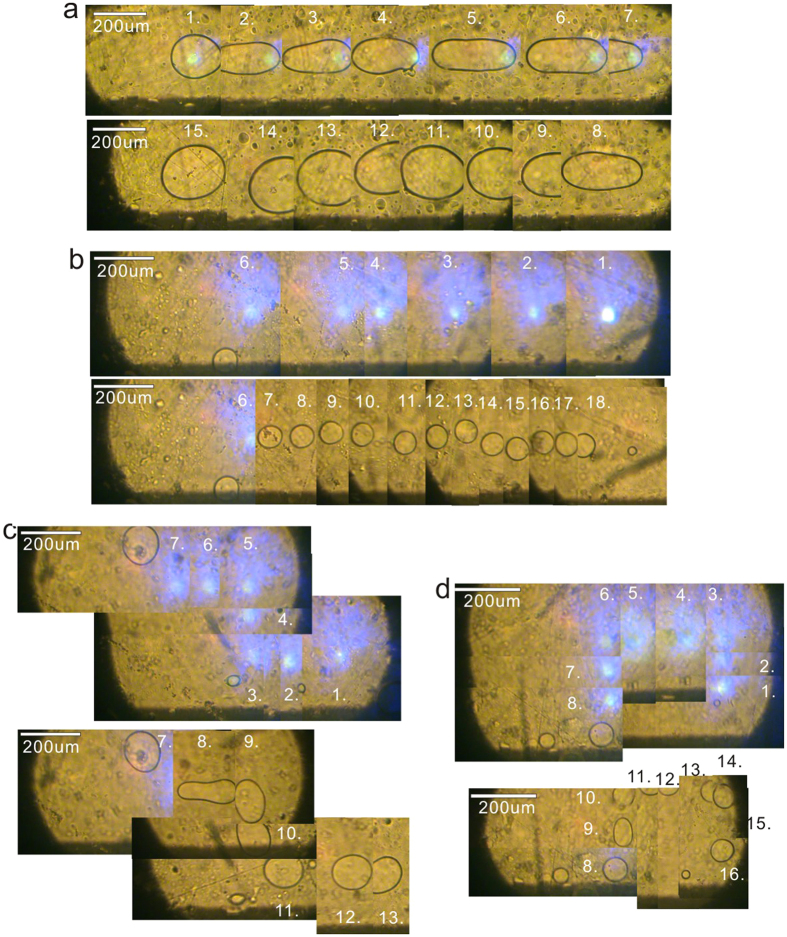
(**a**) The dynamic process of the microdroplet elastically-stretching out and back. (**b**–**d**) The dynamic processes of the microdroplet guiding though the consecutive line-irradiation scheme. In order to show the movement of the scanning laser spot and the trail of the microdroplet more intuitively, many representative frames cut from the original video are pieced together here. In (**a**), the microdroplet location is set as the origin of the line-irradiation and the laser spot later scanned with an increasing speed (from 120 um/s to 210 um/s). In (**b**), the similar scanning is done from a distance and the laser spot is made to approach the microdroplet gradually. In (**c**) and (**d**), the microdroplets are guided in the folding-line trails.

The consecutive line-irradiation scheme is then tried for microdroplet manipulation. In order to show the movement of the scanning laser spot and the microdroplet trail more intuitively, many representative frames cut from the original video are pieced together as presented in Fig. 6. The way we prepare the images here is just like the “Panorama mode” of common digital camera and the numbers in the frames represent the sequence in time.

In Fig. 6(a), the microdroplet location is set as the origin of line-irradiation and the laser spot later scanned with an increasing speed (from 120 um/s to 210 um/s). It is found that the microdroplet is firstly stretched out following the scanning trail but it is then drawn back in the same trail. On the contrary, if we start the similar scanning from a distance and make the laser spot approaching the microdroplet gradually (see Fig. 6(b)), we observe that the microdroplet can be guided by the scanning trail to the origin of line-irradiation. The whole guiding process is smooth without obvious jam and it takes about 10 s to cover 1.7 mm distance. Figure 6(c,d) show the results of folding-line-irradiation scheme and the smooth guiding is also achieved.

The microdroplet manipulation demonstrated above is concerning the motion of a deformable dielectric fluid particle in a nonuniform electrostatic field. The theoretical study about this issue has been done by J.Q. Feng^41^. He showed that the DEP force produced by the nonuniform electrostatic field could induce both deformation and motion of the dielectric fluid particle. Thus, the magnitude order of the electrostatic field required for moving the oil microdroplet should be comparable to that required by the microdroplet generation. The exact amplitude of the electrostatic field could be calculated indirectly from the deformation extent of the oil microdroplet, basing on the relationship between the shape function of fluid particle and the reference electrical field E. Now, the experiments for the systematical investigation of the microdroplet manipulation are still underway.

**Figure 7:**
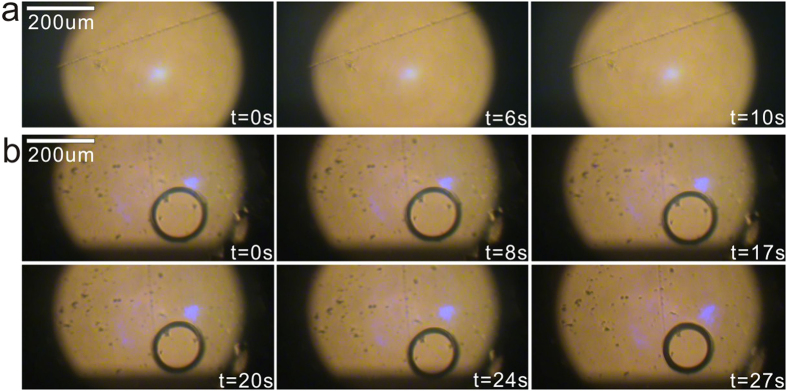
The dynamic processes of the attempted generation (**a**) and manipulation (**b**) for water microdroplet. It can be seen that no water bridge is formed inside the gap (**a**) and no obvious movement of water microdroplet is observed except its slow evaporation due to light-induced thermal effect (**b**).

In addition, oil microdroplets were chosen as the investigation object. However, water microdroplets are more widely used than oil microdroplets in lab-on-chip. Therefore, water microdroplet related studies are necessary from the practice viewpoint. In fact, we have preformed some experiments on water microdroplets and found the manipulation of water microdroplets on naked LN substrates much harder than oil microdroplets. Figure 7 shows the dynamic processes of the attempted generation and manipulation for water microdroplet. One can see that no water bridge is formed inside the gap (see Fig. 7(a)) and no obvious movement of water microdroplet is observed except its slow evaporation due to light-induced thermal effect (see Fig. 7(b)). The discrepant behaviour between water and oil microdroplets is probably due to the higher conductivity and permittivity of water microdroplets. But we have to point out that, after covering LN substrate with a thin super hydrophobic film we found the manipulation efficiency of water microdroplets largely increased. Considering the same liquid nature of oil and water, the preliminary study on dielectrophoretic behaviours of oil microdroplets is beneficial to the following investigation about water microdroplets. The detailed results regarding water microdroplets will be reported in forthcoming papers.

## Conclusions

The behaviours of the dielectric microdroplet under LN-based sandwich configuration are investigated in this paper. It is found that the microdroplet can generate in the form of the liquid bridge inside the LN-based sandwich structure under the action of DEP force, and the dynamic process of microdroplet generation highly depends on the substrate combinations. Dynamic features, including the liquid meniscus profiles and the irradiation-intensity dependences of the time required by the microdroplet generation, are observed for different combinations and studied basing on the electrostatic-field simulations. The volcano-like liquid meniscus profile and the vibration-like liquid movement make the combination of +c surface to +c surface (Case 2) more complicated than the combination of +c surface to −c surface (Case 1). Different kinds of the microdroplet manipulation are also attempted. We suggest that the local DEP force acting on the microdroplet depends on the distribution of the accumulated irradiation dosage. The microdroplet can be step-moved, deformed or patterned by the inconsecutive dot-irradiation scheme, as well as elastically-stretched out and back or smoothly-guided in a designed pass by the consecutive line-irradiation scheme.

## Additional Information

**How to cite this article**: Chen, L. *et al*. Dielectrophoretic behaviours of microdroplet sandwiched between LN substrates. *Sci. Rep*. **6**, 29166; doi: 10.1038/srep29166 (2016).
